# Post-streptococcal glomerulonephritis in Xakriabá indigenous children: case reports

**DOI:** 10.1007/s13730-026-01101-w

**Published:** 2026-04-07

**Authors:** Dilceu Silveira Tolentino Júnior, Hilana Danielle Honorato Veloso, Maelso Bispo de Sousa, Maiele Bispo de Sousa, Roberto Carlos de Oliveira, Eliseu Miranda de Assis

**Affiliations:** 1 Postgraduate Program in Collective Health, René Rachou Institute, Oswaldo Cruz Foundation, Belo Horizonte, MG Brazil; 2 Brejo Indigenous Basic Health Unit, São João das Missões, MG Brazil; 3 University of Brasília, Brasília, DF Brazil; 4 Secretariat for Indigenous Health, Brasília, DF Brazil; 5 Academic Department, Federal Institute of Bahia, Eunápolis, BA Brazil

**Keywords:** PSGN, Impetigo, Indigenous children, Kidney health, Case reports

## Abstract

Post-streptococcal glomerulonephritis (PSGN) is a serious public health problem that mainly affects children. Although rare, the disease affected two children from the Xakriabá indigenous community, being the most common cause of acute nephritis in this age group and potentially leading to irreversible kidney damage if not treated promptly. We report two cases of PSGN in indigenous children that occurred in the village of Brejo, municipality of São João das Missões, Minas Gerais, Brazil. The cases were identified between August and December 2024. All cases were observed in male rural residents, aged between 8 and 10 years. The cause of the disease was a previous cutaneous infection by *Streptococcus pyogenes*, confirmed by titration of type O antistreptolysin. The predominant symptoms were hypertension, facial and lower limb edema, microscopic hematuria, and abdominal pain. All cases were treated in a hospital setting and recovered without complications. We recommend that health teams encourage regular hygiene practices and that the government invest in improvements in environmental sanitation to protect the kidney health of this vulnerable indigenous population.

## Introduction

Post-streptococcal glomerulonephritis (PSGN) is primarily associated with previous skin infections caused by group A streptococci, and is currently observed mainly in economically disadvantaged communities [1]. Streptococcal pyrogenic exotoxin B and the nephritis-associated plasmin receptor are identified nephrotogenic antigens [2]. The pathogenesis of PSGN is multifactorial and may involve the formation of antigen-antibody immune complexes, causing inflammatory damage to the renal glomeruli [3].

PSGN is relatively common worldwide, with multiple outbreaks reported, especially among indigenous peoples of North, Central and South America [4–9] and among Australian Aborigines [10]. In Brazil, although there have been no significant recent outbreaks, PSGN is a concern, mainly in regions with poor sanitary conditions and among children [11]. In Minas Gerais, the second-to-last recorded outbreak of PSGN occurred in the rural area of ​​Nova Serrana between December 1997 and July 1998, caused by the ingestion of unpasteurized milk obtained from cows with mastitis caused by Streptococcus zooepidemicus, with the identification of 253 cases [4]. The last documented outbreak to date occurred in Monte Santo de Minas between December 2012 and February 2013, also caused by Streptococcus zooepidemicus, with 175 confirmed cases, associated with the consumption of milk and dairy products [11].

**Fig. 1:**
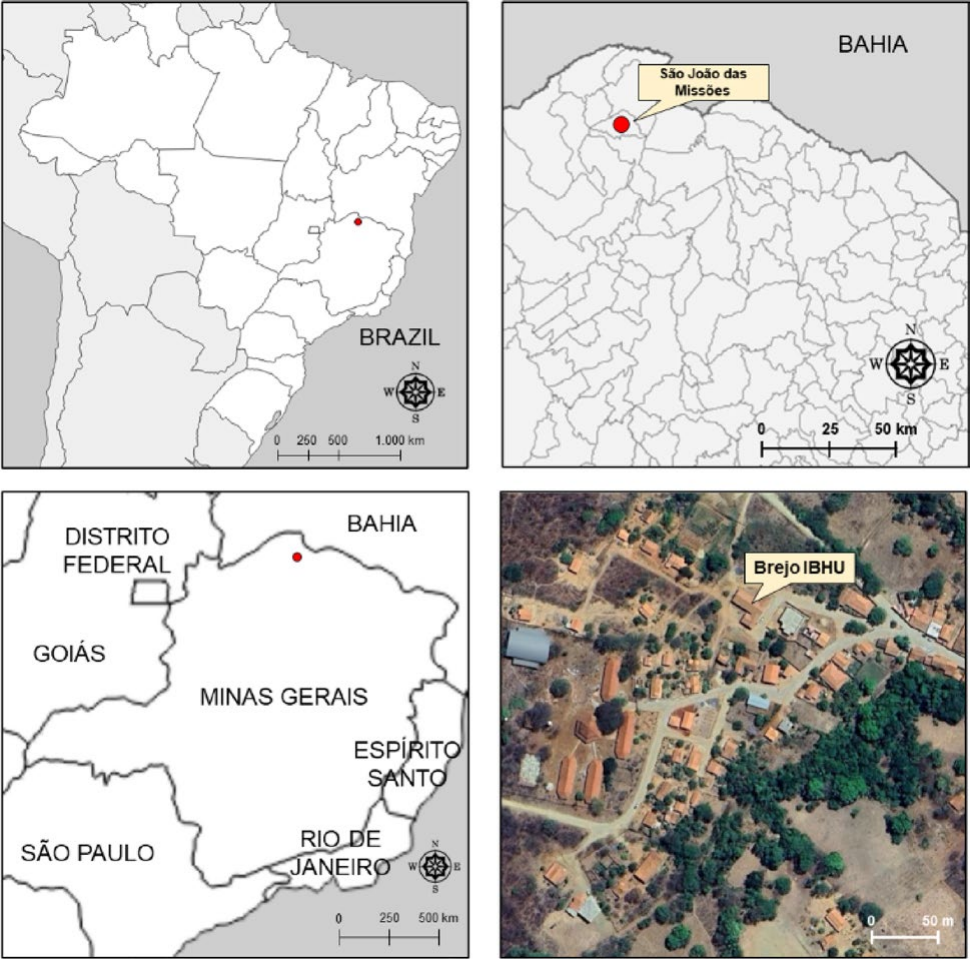
Geographic location of the Brejo Indigenous Basic Health Unit, located in the municipality of São João das Missões, Minas Gerais, Brazil. *Note*: IBHU: Indigenous Basic Health Unit

Between August 5 and December 15, 2024, for the first time, two cases of PSGN occurred in two Xakriabá indigenous children, residents of the village of Brejo (Fig. 1), municipality of São João das Missões-MG, related to previous untreated skin infections by *Streptococcus Pyogenes* [12]. The Xakriabá indigenous people live in one of the municipalities with the lowest human development index in the country, with low life expectancy and high mortality rates from infectious and parasitic diseases [13].

Considering the scarcity of recently published studies on PSGN worldwide, coupled with the rare occurrence of this outcome in Brazilian indigenous populations, we aim to describe the clinical and epidemiological aspects of these cases, presenting a successful experience and the main challenges overcome.

## Case reports

### Case 1

**Fig. 2:**
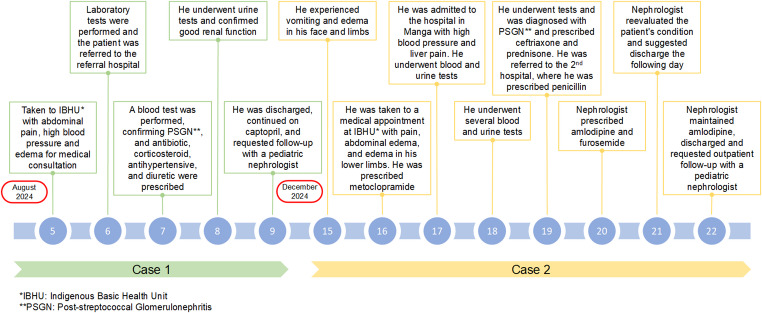
Chronology of post-streptococcal glomerulonephritis cases in Xakriabá indigenous children, São João das Missões, Brazil, 2024

A 10-year-old Indigenous boy, from the Xakriabá ethnic group, from the village of Brejo, São João das Missões, a student, with no history of personal, family, or psychosocial comorbidities, except for the presentation of untreated impetigo lesions 2 weeks prior. On August 5th, he consulted at the Indigenous Basic Health Unit (IHBU) complaining of dyspnea on exertion, nausea, abdominal pain, blood pressure of 150 × 100 mmHg, cough, fever, periorbital edema, facial jaundice, dehydrated mucous membranes, bilateral crackling rales, and increased heart sounds. On August 6th, after laboratory tests, he was referred to the Janaúba hospital with the following results: antistreptolysin type O (< 200 IU/mL), hematocrit (34.80%); hemoglobin (Hb) 11.40 g/dL; Red blood cells (4.05 million/mm^3^), white blood cells (7300/mm^3^), urea (14.00 mg/dL), creatinine (0.60 mg/dL), ALT (35 U/L) and AST (28 U/L). The urine test revealed a light yellow color, semi-turbid appearance, presence of hemoglobin (++), an average of 20 red blood cells per field, 2 medium epithelial cells per field, without proteins or leukocytes. On August 7th, the dose of antistreptolysin O (> 200 IU/mL) was titrated, confirming the diagnosis of post-streptococcal glomerulonephritis (PSGN). Intramuscular injection of benzathine penicillin (1,200,000 IU, single dose), captopril (1 oral tablet, 12.5 mg, twice daily), furosemide (1 oral tablet, 0.5 mg/kg, twice daily), and prednisone (1 oral tablet, 1 mg/kg/day) were prescribed. On August 8th, urinalysis was performed, showing potassium (4.4 mEq/L), albumin (4.4 mg/g), and proteinuria (190 mg/24 h), demonstrating good renal function. On August 9th, after clinical follow-up, the nephrologist discontinued the use of prednisone and decided to discharge the child, maintaining captopril and requesting short-term outpatient follow-up with a pediatric nephrologist for complementary tests (Fig. 2). On August 29th, the test results were re-evaluated by the IHBU physician, with the following results: urea (11.00 mg/dL), creatinine (0.50 mg/dL), proteinuria of 100 mg/24 h, and absent hemoglobinuria.

Regarding socio-environmental conditions, the dwelling is an adobe house with four rooms, where the child shares the space with four other people; there is no bathroom. The residence has electricity, piped water (untreated) from an artesian well, but no sewage system. Garbage is not collected but burned near the residence. The mother is a homemaker, the father is a farm worker, and they do not have a fixed salary (Table 1); their main income is a government subsidy of USD 142.50 per month.

**Table 1:** Comparison of social and environmental factors in two cases of post-streptococcal glomerulonephritis in Xakriabá children, 2024

Dimension	Factor	Case 1	Case 2	Implications
Social	Socioeconomic condition	High social vulnerability	High social vulnerability	Associated with a higher risk of untreated infections
	Education level of caregivers	Low level of formal education	Medium level of formal education	Makes early recognition of symptoms difficult
	Access to health information	Limited	Limited	Reduces adherence to preventive measures
	Cultural barriers	Present	Present	They hinder communication with health services
	Community organization	Intense collective living	Intense collective living	Facilitates the circulation of streptococci
Environmental	Housing conditions	Medium household density	Medium household density	Increases skin transmission
	Basic sanitation	Absent or inadequate	Absent or inadequate	Promotes skin infections
	Water supply	Irregular or untreated	Irregular or untreated	Increases the risk of impetigo
	Environmental hygiene	Structural limitations	Structural limitations	Maintains infectious foci
	Weather conditions	Hot and dry weather	Hot and dry weather	May worsen skin infections
	Presence of animals	Animals near residences	Animals near residences	Potential indirect environmental contamination

### Case 2

An 8-year-old Xakriabá indigenous boy from the village of Brejo, São João das Missões, a student, with no history of personal, family, or psychosocial comorbidities. Evaluated by the health team, crusted lesions were identified on his lower limbs for eight days, which worsened after the appearance of edema in the limbs. Similar crusted lesions were also identified on the lower limbs of his brother. On December 15th, he presented with facial and limb edema, as well as vomiting. On December 16th, he was seen at IBHU with pain, abdominal edema, and edema in the lower limbs. Metoclopramide (5 mg, 26 drops orally, 3 times a day) was prescribed. On December 17th, he was admitted to the hospital in Manga—MG with blood pressure of 150 × 90 mmHg, a palpable and painful liver, and no alteration in diuresis. On December 17th, blood tests were performed: antistreptolysin type O (> 200 IU/mL), hemoglobin (Hb) of 10.6%, hematocrit of 31.7%, total leukocytes of 11,000/mm^3^, creatinine of 1.0 mg/dL and urea of ​​89 mg/dL; urinalysis: absence of proteins, hemoglobin present, 6 pyocytes per field and fields full of red blood cells. On December 18th, the following tests were performed: hemoglobin (Hb) (11%), hematocrit (Ht): 31.7%, platelets (295,000 µL), creatinine (0.92 mg/dL), urea (63 mg/dL), high-density lipoprotein (HDL) of 73 mg/dL, and low-density lipoprotein (LDL) of 55.40 mg/dL. The urinalysis showed positive hemoglobin (3+), 25 red blood cells per field, microalbiminuria (71.2 mg/dL), and albumin (86.5 mg/g). On December 19th, a renal and urinary tract ultrasound was performed, with normal findings and mild ascites. The patient received ceftriaxone (IV), 50 mg/kg/day, prednisone (1 tablet orally, 1 mg/kg/day), and furosemide (1 tablet orally, 0.5 mg/kg, twice daily). On December 19th, the patient was referred to the hospital in Montes Claros, where benzathine penicillin (1,200,000 IU, IM, single dose) and furosemide (1 tablet orally, 0.5 mg/kg, twice daily) were prescribed. On December 20th, a nephrologist discontinued prednisone and prescribed amlodipine (5 mg orally, 0.5 tablet in the morning) and furosemide (20 mg orally, 1 tablet in the morning), suggesting discharge with home medication. On December 21st, the nephrologist reassessed the patient and found no complications. On December 22, the nephrologist maintained the amlodipine (2.5 mg orally/day) and discharged the patient with a referral to a pediatric nephrologist (Fig. 2). He had his last follow-up appointment with a pediatric nephrologist on January 20, 2025, with serum creatinine results of (0.47 mg/dL), serum urea of ​​(40 mg/dL), and no proteinuria or hemoglobinuria.

Regarding socio-environmental conditions, he lives in a brick house with 4 rooms, where he shares the space with 4 other people. The residence has electricity, a bathroom, untreated piped water from an artesian well, and no sewage system. Garbage is not collected but is burned near the residence (Table 1). His mother is divorced, a teacher, and has a monthly income of approximately USD 471.78.

## Discussion

PSGN can develop 2–6 weeks after an untreated streptococcal infection, due to a delayed immune response to certain strains of streptococcal bacteria that cause impetigo [14]. Without treatment, resolution can take 30 days [15] and infection of all other family members can occur in 21 days [16]. In addition to PSGN, impetigo can also cause acute rheumatic fever, chronic kidney disease, and rheumatic heart disease [17]. As with Australian Indigenous children, who have the highest rate of impetigo in the world (49%) [18], Xakriabá Indigenous children also have a high prevalence of this dermatosis, sharing environmental, socioeconomic, and health factors that make these populations more vulnerable to infection and its complications [19].

Based on these two cases alone, it is not possible to say that children are vulnerable to PSGN in the Xakriabá territory, since two isolated cases do not characterize high incidence or endemicity, and may represent sporadic, underreported events or be associated with a localized outbreak of streptococcal infection. To support the claim that this disease is common in this region, an active surveillance system would be necessary, with availability of longitudinal epidemiological data and historical series of notifications [20].

Overcrowding in homes [21] and lack of hygiene are conditions that increase the risk for the development of PSGN [19], especially in indigenous communities. In the Xakriabá community, each house houses one to 15 people, who share the same sleeping space. 29% of the houses do not have electric lighting. It should be noted that, of the 925 houses (75.57%), there is no bathroom or sanitary facilities. Most of the Xakriabá population still uses water from streams, rivers, wells, cisterns, dams, lagoons or water distributed by transport vehicles [19]. Although there is a basic indigenous health unit in the Brejo community with a nursing team on duty, the unit faces structural and logistical challenges, such as high turnover of health professionals, lack of paved roads that allow access to the community, distance of more than 22 km from the IBHU to the municipal headquarters and considerable distance from the IBHU to the nearest reference hospital units (approximately 46 km to Manga, 190 km to Janaúba and 277 km to Montes Claros), considered the only resources for diagnostic and therapeutic support for the most serious cases [13].

Other important predisposing factors for the indigenous population are the association of *S. pyogenes*, scabies, endoparasitoses and childhood malnutrition, which can trigger streptococcal outbreaks that are difficult to control, explaining the higher incidence of PSGN in poorer regions of the world [21]. It is also believed that genetic factors predispose to the disease, since almost 40% of patients with PSGN have a positive family history. However, to date, no specific gene has been identified as causing PSGN [22]. Untreated infections by group A beta-hemolytic streptococci are a triggering factor for individuals with genetic susceptibility to develop autoimmune diseases such as GNPE [22, 23].

The hospitalization of Xakriabá children for an average of 4 days was necessary for adequate diagnostic support, which was similar to the average hospital stay of Nepalese children with PSGN, which was 4–8 days [24]. However, treatment with antibiotics and immunosuppressants remains controversial [24]. In both cases, blood pressure improved as fluid retention resolved, generally between 1 and 2 weeks after the onset of symptoms, with clinical control and, when necessary, with the use of antihypertensives and fluid/sodium restriction [25]. The diuretic phase began after 1 week, marking the recovery of renal function, when there was a progressive increase in urine volume and a reduction in edema, coinciding with overall clinical improvement and the gradual normalization of laboratory parameters [26].

Although bacterial culture of purulent lesions is the most important diagnostic method to demonstrate previous streptococcal infection, in our sample the diagnosis was determined by measuring the type O antistreptolysin (ASO) titer, accompanied by tests that corroborated the diagnosis, such as ultrasound, blood and urine tests. The failure to perform bacterial culture in purulent lesions and serum C3 levels in indigenous populations does not stem from a single factor, but from a context of structural and programmatic vulnerability, in which immediate clinical care prevails over etiological confirmation [27]. Overcoming these barriers requires strengthening the laboratory network, qualifying the teams, adequate logistics and an intercultural approach, as well as integration between care and epidemiological surveillance [28]. The laboratory tests performed were similar to those performed in a remote indigenous community in Canada, where urea, creatinine, complete blood count and ASO titration were also performed [29].

The distance of the village from health services, coupled with transportation difficulties, contributed to delays in clinical assessment [30]. Added to this are limitations in the initial recognition of the severity of skin lesions, both by families and by professionals unfamiliar with local living conditions, as well as the normalization of skin infections as common childhood events [31].

We observed that, due to the scarcity of nephrologists in this region, the initial treatment of cases was controversial, using prednisone prescribed by general practitioners instead of prescribing only diuretics and antihypertensives [32]. With the lack of nephrologists in the region, local managers attempt to manage complex cases of renal patients without the necessary specialization, which can result in less effective or unsafe management [33].

Specialized assessment contributed to guiding timely therapeutic interventions and reducing the risk of chronic sequelae. In indigenous contexts, where access to specialized health services is often limited and socio-environmental conditions can favor the occurrence and recurrence of streptococcal infections, the role of the nephrologist is also essential for the development of prevention strategies [29].

In these cases of PSGN, prevention and control measures are crucial to minimize the spread of the disease and treat existing cases [34]. The local health team adequately monitored close and family contacts to identify suspected cases, initiating immediate treatment of impetigo cases to prevent possible PSGN outcomes. In addition, other important measures adopted included the early diagnosis and treatment of streptococcal infections similar to the actions taken in response to outbreaks of pediatric cases in the region [12].

The two cases of PSGN occurred in a territory marked by high social vulnerability, in which social and environmental factors acted convergently to increase the risk of streptococcal infections and their complications [35]. In both cases, precarious housing conditions, limitations in access to basic sanitation, and barriers to health care were observed, creating a scenario conducive to the transmission of skin infections. However, the first case presented a higher degree of exposure to these determinants, evidenced by even more deficient hygiene conditions, especially the absence of a bathroom in the residence, in addition to lower monthly family income, which may have intensified individual vulnerability and contributed to a greater risk of developing post-infectious complications [35].

The lesson learned is that Indigenous health teams need to adapt to coordinate control and prevention measures with their own resources, ensuring a faster response aligned with the needs of this Indigenous community. The collection and recording of epidemiological information by the team remains limited, hindering the analysis and planning of future actions. These experiences demonstrate that, despite advances, there are still challenges to overcome to ensure increasingly assertive case management.

We hope that this case report will serve as a warning for health authorities to become more sensitive to the health of Brazilian Indigenous children, providing them with rapid access and improving the quality of specialized care and basic sanitation in their communities, aligning with the goal of reducing the burden of kidney disease in underserved populations.

Local health teams should strive to identify skin infections more quickly and treat them appropriately to prevent them from progressing to PSGN. In addition, awareness campaigns should be implemented in Indigenous communities, encouraging the search for early medical care upon the onset of symptoms.

Groups in vulnerable situations, marked by insufficient sanitary conditions, nutritional limitations, low levels of education, and difficulties in accessing structured health services, are more prone to maintaining contexts that favor the emergence of diseases such as those observed in these two cases. The association between *S. pyogenes* and other adverse conditions may contribute to the occurrence of streptococcal outbreaks, although fortunately this was not observed in this report. In this context, encouraging regular hygiene practices and improving sanitation conditions in communities are important prevention strategies, with a positive impact on protecting the renal health of these populations.

## Data Availability

Data is provided within the manuscript.

## Abbreviations

A/U: Arbitrary units
ALT: Alanine aminotransferase
ASO: Antistreptolysin O
AST: Aspartate aminotransferase
IBHU: Indigenous Basic Health Unit
IM: Intramuscular route
IU: International units
mEq: Milliequivalent
mm: Millimeter
PSGN: Post-streptococcal glomerulonephritis
